# Dendrite-Free Li Metal Plating/Stripping Onto Three-Dimensional Vertical-Graphene@Carbon-Cloth Host

**DOI:** 10.3389/fchem.2019.00714

**Published:** 2019-10-25

**Authors:** Congcong Yan, Tingting Xu, Caiyun Ma, Jinhao Zang, Junmin Xu, Yumeng Shi, Dezhi Kong, Chang Ke, Xinjian Li, Ye Wang

**Affiliations:** ^1^Key Laboratory of Material Physics of Ministry of Education, School of Physics and Engineering, Zhengzhou University, Zhengzhou, China; ^2^International Collaborative Laboratory of 2D Materials for Optoelectronics Science and Technology of Ministry of Education, Institute of Microscale Optoelectronics, Shenzhen University, Shenzhen, China; ^3^School of Electrical and Electronic Engineering, Nanyang Technological University, Singapore, Singapore

**Keywords:** Li metal anode, three-dimensional VG/CC, dendrite-free, excellent electrochemical performance, long cycle stability

## Abstract

Lithium metal is deemed as an ideal anode material for next-generation lithium ion batteries (LIBs) due to its high specific capacity and low redox potential. However, uncontrolled lithium dendrite formation during electrochemical deposition leads to a low Coulombic efficiency and serious safety issues, dragging metallic lithium anodes out of practical application. One promising strategy to suppress lithium dendrite issues is employing a three-dimensional host with admirable conductivity and large surface area. Herein, a vertical graphene nanosheet grown on carbon cloth (VG/CC) synthesized is adopted as the Li deposition host. The three-dimensional VG/CC with a large surface area can provide abundant active nucleation sites and effectively reduce the current density, leading to homogeneous Li deposition to overcome the dendrite issue. The Li@VG/CC anode exhibits a dendrite-free morphology after a long cycle and superior electrochemical performance to that of planar Cu current collector. It delivers a small voltage hysteresis of 90.9 mV at a high current density of 10 mA cm^−2^ and a Coulombic efficiency of 99% over 100 cycles at 2 mA cm^−2^. Our results indicate that this all-carbon-based nanostructure host has great potential for next-generation Li metal batteries.

## Introduction

Lithium-ion batteries (LIBs) have dominated the energy storage market owing to its high energy density, long cycle life, absence of memory effect and low pollution (Chu and Majumdar, 2012; Chu et al., 2016; Cheng et al., 2017; Lim et al., 2019a). However, the energy density of currently commercial LIBs needs to be further improved (Du et al., 2016). One of the solutions is exploring high-capacity anode materials, as the theoretical capacity of commercial graphite is only 372 mAh g^−1^ (Bruce et al., 2012; Cheng et al., 2016b). Among various advanced anode materials, lithium metal has a high theoretical capacity (3,860 mAh g^−1^) and low electrochemical potential (−3.04 V vs. the standard hydrogen electrode; Cheng et al., 2016a). Therefore, lithium metal is considered to be one of the most promising anode materials (Xu et al., 2014; Zhang et al., 2016). In particular, lithium metal is also widely employed as the anode of other high-energy density batteries such as Li-S and Li-O_2_ batteries (Cheng et al., 2015; Grande et al., 2015; Ma et al., 2015; Manthiram et al., 2015; Huang et al., 2018). Therefore, it is timely and urgent to develop high performance lithium metal anode (Huang et al., 2014). However, the formation of unstable solid electrolyte interphase (SEI) due to lithium metal high electrochemical reactivity and infinite volume change upon repeated plating/stripping causes the severe uncontrolled lithium dendrite growth, leading to a low Coulombic efficiency and internal short circuit even severe safety issue (Lim et al., 2019b).

In order to tackle these issues, great efforts have been devoted to depress the lithium dendrite formation: optimization of the electrolytes with additives, artificial SEI layer, nanoscale interface engineering, etc. (Liu B. et al., 2018). A 3D electrode host with a large surface area is demonstrated as an effective strategy to suppress the dendrite formation due to the reduced current density (Li et al., 2017; Ma et al., in press). Graphene is a kind of carbon materials with a single/few layers of carbon atoms arranged in a hexagonal lattice (Deng et al., 2014; Lin et al., 2016). As a two-dimensional material with many merits, horizontal graphene has been used as the acritical SEI layer and 3D host of lithium metal anode (Chen et al., 2018; Liu S. et al., 2018; Wu et al., 2018; Li et al., 2019). Different from the traditional graphene grown/stacked in the horizontal direction, vertical graphene (VG) is a type of graphene grown in vertical direction, naturally hosting the lithium metal. For example, 3D vertical graphene nanowalls (VGN) grown on nickel (Ni) foam (VGN/Ni) by mesoplasma chemical vapor deposition (MPCVD) are employed as the lithium metal host (Ren et al., 2018). The symmetric battery VGN/Ni@Li stably cycles more than 2,000 h at a current density of 0.5 mA cm^−2^ (Ren et al., 2018). Vertical graphene nanosheets grown on copper foam (Cu@VG) prepared by plasma-enhanced chemical vapor deposition (PECVD) is also employed as the lithium metal host (Hu et al., 2019). The assembled Cu@VG@Li battery can stably cycle for 100 h at a current density of 3 mA cm^−2^ with a capacity of 3 mAh cm^−2^ (Hu et al., 2019). VG on Ni/Cu foam has a large surface area, which provides plenty of nucleation centers for the lithium metal and effectively reduces the current density, resulting in an improved electrochemical performance. All the previous reports employed metal (Ni or Cu) as the scaffold to support the graphene. Compared with Ni or Cu foam, carbon scaffold has the merits of being lightweight, flexible, low-cost and renewable (Xu et al., 2017; Li et al., 2018b; Jiang et al., 2019). Carbon-based VG host is of great interest for flexible, low-cost and sustainable lithium metal batteries. However, there have been no reports of carbon as the scaffold to support VG as the lithium metal host until now.

**Figure 1:**
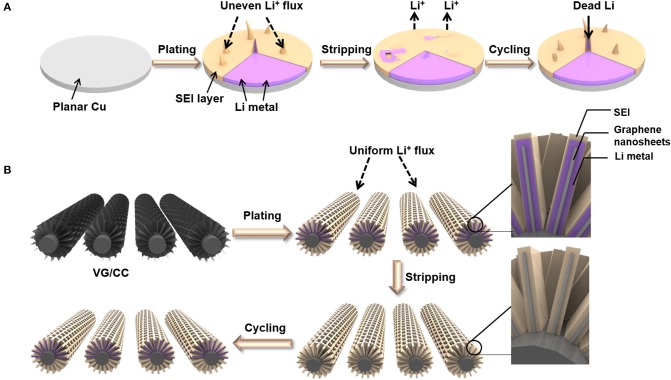
Schematic illustration of the Li plating/stripping process on **(A)** planar Cu and **(B)** VG/CC.

Herein, we report a metal-free 3D interconnected VG grown on the surface of carbon cloth (VG/CC) as the host of lithium metal to form a dendrite-free nanostructure. With the porous 3D structure, VG/CC allows controllable and dendrite-free lithium metal plating/stripping (Figure 1). The volume change during the plating/stripping process is also well-adapted (Wang et al., 2018b). The three-dimensional structure increases the specific surface area relative to the planar Cu, reducing the local current density, effectively inhibiting dendrite growth, and improves long-term cycle stability and rate performance. The battery can stably cycle with a long lifespan over 500 cycles at the current density 10 mA cm^−2^ and a high Coulombic efficiency of >99%. In order to explore the real application, a full battery composed of Li@VG/CC as the anode and the LiCoO_2_ (LCO) as the cathode exhibits a capacity of 133 mAh g^−1^ at 0.2 C.

## Materials and Methods

### Synthesis of VG/CC

The synthesis process of the VG/CC can be found in previous reports (Wang et al., 2016; Ghosh et al., 2017). In brief, VG grown on CC was synthesized by a plasma-enhanced chemical vapor deposition (PECVD) and CC as substrate was placed in the center of the plasma-generating region. The atmosphere was CH_4_/H_2_ (40/60%), the pressure was controlled to 25 Pa and the RF power was 1,000 W. The growth was 20 min to synthesis VG/CC with a substrate temperature of 700°C.

### Materials Characterizations

The morphology of the as-prepared VG/CC was characterized by field emission scanning electron microscopy (JEOL, JSM-6700F) and high-resolution transmission electron microscopy (HRTEM, JEOL, JEM-2100). The crystal structure of the sample was performed by X-ray diffraction with Cu Kα radiation (XRD SmartLab 3 KW). Raman spectra were carried out by confocal Raman spectroscopy (HORIBA, LabRAM HR Evolution).

### Electrochemical Deposition to Fabricate Li@VG/CC Anode and Electrochemical Performance Evaluation

The Li@VG/CC was fabricated by a standard electrochemical deposition process. In brief, the prepared VG/CC and the lithium foil were used as the working and counter electrode, respectively. Two pieces of membrane (Celgard 2400) were used as the separator, and 1 M lithium bis(trifluoromethane)sulfonylimide (LiTFSI) in 1,3-dioxolane: 1,2-dimethoxyethane binary solvent (DOL:DME, 1:1 by volume) with 1% LiNO_3_ (stabilizer) was used as the electrolyte. All of the above components were assembled into CR2032-type coin cells in an argon-filled glove box (<0.1 ppm of oxygen and water) for the next step of the electrochemical deposition process. The metallic Li was plated onto the substrate of VG/CC and planar Cu by a Neware battery discharge/charge equipment. All cells were cycling at 0–1 V (vs. Li^+^/Li) at 50 μA five times to stabilize SEI film and remove surface contamination. For the Coulombic efficiency measurement, 1 or 2 mAh cm^−2^ capacity was plated on VG/CC and then recharged to 1 V at current densities of 1 or 2 mA cm^−2^. The Coulombic efficiency was evaluated by the stripping-capacity/plating-capacity. For long-term constant current discharge/charge tests, all batteries were plated with a constant current capacity of 1 mAh cm^−2^ and stripped up to 1 V at a current density of 1, 2, and 10 mA cm^−2^. Electrochemical impedance spectroscopy (EIS) was measured by an electrochemical workstation (VMP3, Bio-logic) after various electrochemical cycles by applying an alternating voltage of 10 mV over the frequency ranging from 10^5^ to 10^−2^ Hz. The control sample was Li deposited onto a piece of two-dimensional planar Cu, all deposition processes and evaluation processes were the same as that of Li@VG/CC.

For full battery testing, commercial LiCoO_2_ was used as the positive electrode. A total of 80% LiCoO_2_, 10% polyvinylidene fluoride (PVDF), and 10% carbon black were thoroughly ground in air until being homogeneously mixed. Then, NMP was added dropwise to form the slurry, then coated onto an Al foil. The resulting electrode sheet contains ~2 mg cm^−2^ of LiCoO_2_. For full battery long-term cycling, 2 mAh cm^−2^ Li metal was deposited onto VG/CC or planar Cu to match the LiCoO_2_. The electrolyte was 1 M LiPF_6_ in ethylene carbonate (EC) and diethyl carbonate (DEC) (1:1 by volume). The voltage test range was 2.5–4.5 V.

## Results and Discussions

**Figure 2:**
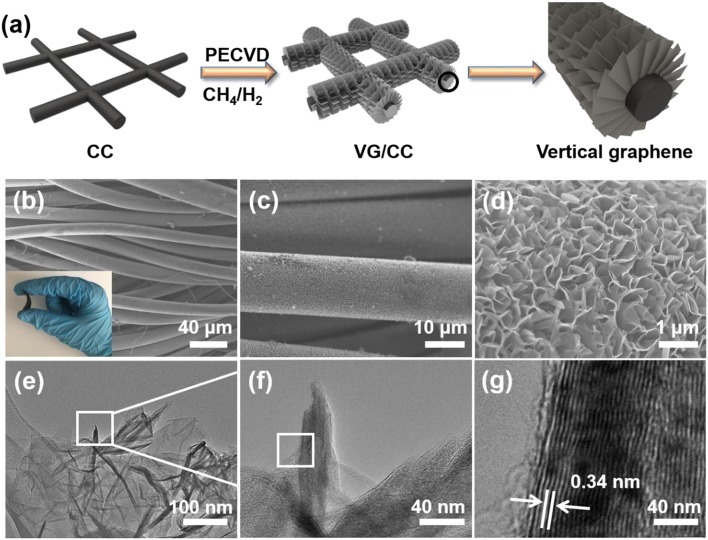
**(a)** Schematic illustration of the synthesis process of the VG/CC by PECVD. **(b)** Low, **(c)** high, and **(d)** higher magnification SEM images of VG/CC. Insets: photograph of bent VG/CC, demonstrating the good mechanical strength of VG/CC. TEM image of VG/CC at **(e)** low and **(f)** high magnifications, **(g)** HRTEM images of VG/CC.

The synthesis process of VG/CC is described in detail in Figure 2a. Vertically grown graphene nanosheets on the surface of the CC are synthesized by a PECVD (Ghosh et al., 2017). CC consists of interconnected carbon nanofibers with good flexibility, strong mechanical strength and high electron transport capability (Figure S1; Tian et al., 2018a). However, CC is lithiophibic with limited surface area (Liu F. et al., 2019). After PECVD process, VG nanosheets are uniformly grown on the surface of CC (Figures 2b–d). The synthesized VG/CC exhibits flexible property as shown in the inset of Figure 2b. The VG grown on the vertical direction may be attributed to the localized electrical field and the plasma/reaction process (Wang et al., 2016). There are plenty of pores/voids between the VG nanosheets, leading to the increased surface area compared to the planar Cu (Figure S2). A tip of VG is characterized by transmission electron microscopy (TEM) (Figures 2e,f). The interplanar spacing of the sample is 0.34 nm, corresponding to the (002) crystal planes of graphene as shown in Figure 2g.

**Figure 3:**
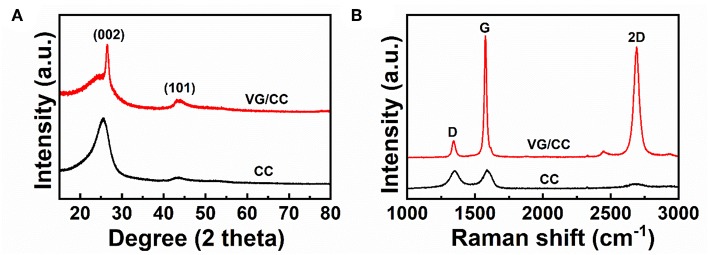
**(A)** XRD patterns and **(B)** Raman spectra of the VG/CC and CC.

The crystal structure is performed by X-ray diffraction (XRD) as shown in Figure 3A. There are two peaks centered at 26 and 42°, corresponding to (002) and (101) planes of the graphitic carbon, respectively (Huang et al., 2019). Raman spectra of the samples are shown in Figure 3B. There are three peaks located at 1,350, 1,590, and 2,690 cm^−1^, which can be attributed to D, G and 2D peaks of graphene, respectively (Cho et al., 2018; Meng et al., 2019). The D and G peaks are corresponding to the disorder carbon atoms in the hexagonal graphitic network and the in-plane vibrational mode of sp2-bonded carbon atoms, respectively (Zhang et al., 2018; Hu et al., 2019). Thus, the D peak indicates there are plenty of defects in the VG/CC nanostructures, which are the nucleation sites for lithium metal plating according to a previous study (Wang et al., 2017). Introduced defects in graphene reduce the lithium metal nucleation energy barrier (Tian et al., 2017; Jiang et al., 2019). In addition, a strong G peak and a high I_G_/I_D_ (6.78) indicate that the graphene sheets have a high degree of graphitization with good electronic conductivity (Kong et al., 2019).

**Figure 4:**
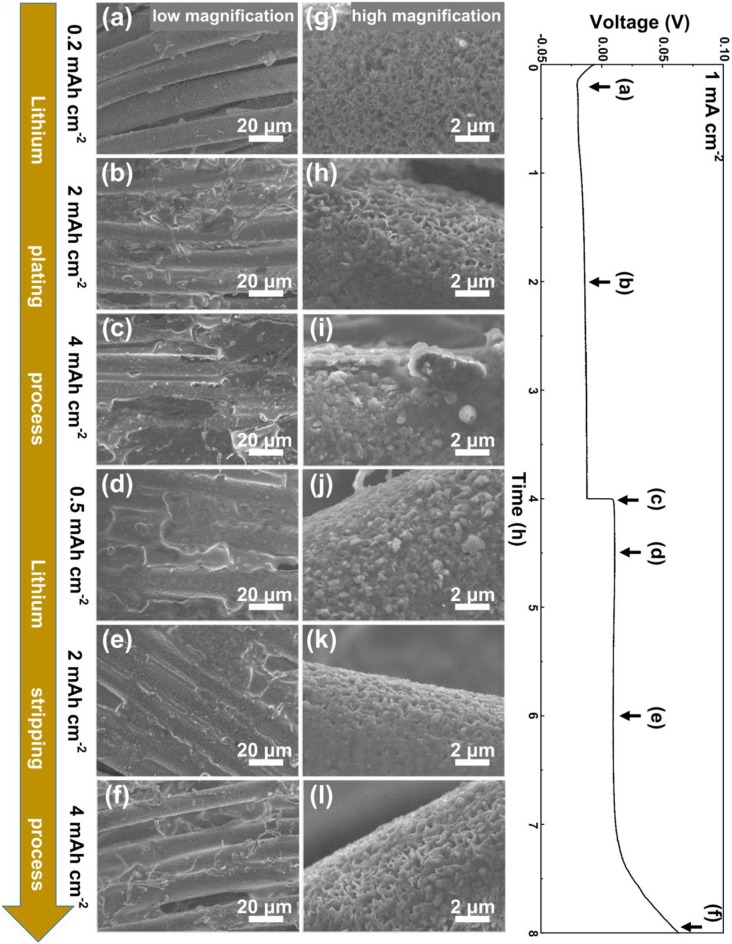
Morphological evolution of Li metal plating and stripping on VG/CC. SEM images of VG/CC electrode after being plated at a capacity of **(a,g)** 0.2 mAh cm^−2^ **(b,h)** 2 mAh cm^−2^, and **(c,i)** 4 mAh cm^−2^, and then stripped **(d,j)** 0.5 mAh cm^−2^, **(e,k)** 2 mAh cm^−2^, and **(f,l)** 4 mAh cm^−2^ at a current density of 1 mA cm^−2^.

In order to investigate the plating/stripping behavior of lithium metal on VG/CC, an electrochemical deposition cell is assembled with VG/CC as the working electrode to electrodeposit lithium metal and lithium foil as the counter electrode to provide the lithium source. The morphology evolution of the VG/CC during the Li metal plating/stripping process is performed by an *ex-situ* SEM method. As shown in Figure 4a, when initial plated capacity reached up to 0.2 mAh cm^−2^, metallic lithium is nucleated and uniformly distributed on VG sheets without obvious protuberance. From the corresponding higher magnification SEM image (Figure 4g), the vertically distributed graphene nanosheets can still be clearly seen. With the capacity increased to 2 mAh cm^−2^ (Figure 4b), the VG nanosheets are still visible and the deposited lithium metal fills the pores and starts to cover the VG surface (Figure 4h). It is also noted that the plated Li metal is prone to horizontally expand from the VG nanosheets to the micro-scale voids between the carbon fibers. This may be owing to the confinement effect due to the nanosheets-reinforced nanostructure (Hao et al., 2016). The excess lithium metal is held in the pores of VG/CC structures. The same phenomena have been observed in nanowire-Cu foam as the Na metal host (Wang et al., 2018a). With further plating capacity increased to 4 mAh cm^−2^ (Figure 4c), the graphene nanosheets are almost fully filled with metallic lithium (Figure 4i). Uniformly small size lithium deposits without dendrites are observed, indicating a uniform lithium ion plating process. Moreover, the thickness of VG/CC electrode is increased from 400.00 μm (before) to 412.50 μm (after Li deposition with a capacity of 4 mAh cm^−2^) (Figures S3a,c). An enlarged view to show the thickness of VG is changed from 993.75 nm (before) to 1,312.50 nm (after Li deposition with a capacity of 4 mAh cm^−2^) (Figures S3b,d). When the lithium is gradually stripped from VG/CC, the lithium metal in pores and voids starts to fade and the VG nanosheets are gradually exposed. When stripping capacity reaches to 0.5 mAh cm^−2^ (Figure 4d), lithium metal begins to fade but still fully covers the surface of the sample (Figure 4j). With the stripping capacity reaching 2 mAh cm^−2^ (Figure 4e), and the graphene nanosheets start to be exposed due to the fade of lithium metal (Figure 4k). With further stripping up to 4 mAh cm^−2^ (Figure 4f), the potential reaches up to 64 mV, and the graphene nanosheets are completely revealed (Figure 4l), suggesting the high reversibility of VG/CC. In contrast, the plating/stripping behavior of planar Cu is shown in Figure S4. When plating capacity reaches to 0.5 mAh cm^−2^, small lithium particles start to nucleate on Cu foil. With the plating capacity increasing from 0.5 to 4 mAh cm^−2^, metallic lithium particles grow bigger and longer, and finally grow into moss tree-like lithium dendrites with a diameter of ~10 μm (Figure S4i). During the stripping process, the lithium dendrite gradually becomes smaller. When the stripping capacity reaches 3.9 mAh cm^−2^, the potential is increased to 500 mV, the reason is owing to all electrically connected lithium is fully stripped. Therefore, 0.1 mAh cm^−2^ lithium is irreversible. However, plenty of microdots (indicated by the red circles) and films can be found on the surface of copper foil, corresponding to the dead lithium dendrite and the SEI film (Cheng et al., 2018; Li et al., 2018a).

**Figure 5:**
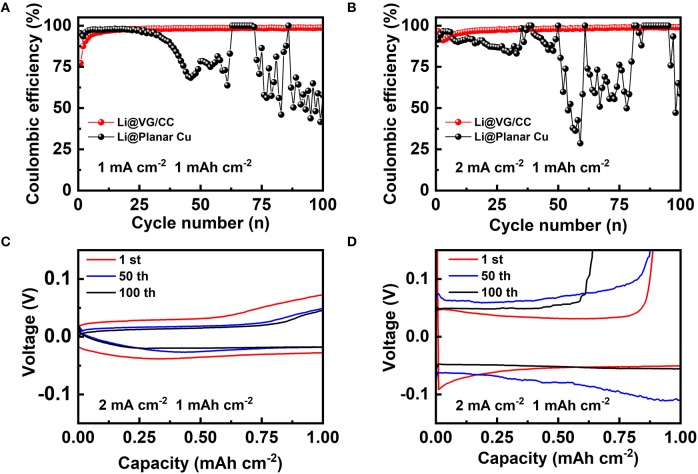
Coulombic efficiency of the VG/CC (red), planar Cu (black) with a cycling capacity of 1 mAh cm^−2^ at a current density of **(A)** 1 mA cm^−2^ and **(B)** 2 mA cm^−2^. Voltage-capacity curves of the **(C)** VG/CC and **(D)** planar Cu after various cycles with a capacity of 1 mAh cm^−2^ at a current density of 2 mA cm^−2^.

Coulombic efficiency is a critical parameter to evaluate the reversibility of the metallic lithium plated into the host. In our work, Coulombic efficiency is measured at different current densities 1 and 2 mA cm^−2^ as shown in Figure 5. The Coulombic efficiency of VG/CC is increased in the first several cycles and then stabled >99% after 20 cycles at 1 and 2 mA cm^−2^. However, the Coulombic efficiency of planar Cu first decreased quickly and fluctuated at all measured conditions. For example, at a capacity of 1 mAh cm^−2^, the Coulombic efficiency of planar Cu is higher than 98% at a current density of 1 mA cm^−2^ from the 1st to 25th cycle, and then dropped quickly and fluctuated (Figure 5A). It is more unstable at a current density of 2 mA cm^−2^ with the capacity of 1 mAh cm^−2^ (Figure 5B). In comparison, the Coulombic efficiency of VG/CC gradually stable at 99% at current densities of 1 mA and 2 mA cm^−2^ (both at 1 mAh cm^−2^). It is worth mentioning that the Coulombic efficiency of VG/CC is also higher than 97% at 1 mA cm^−2^ (2 mAh cm^−2^) (Figure S5). The inferior Coulombic efficiency of the planar Cu is due to the uneven Li nucleation and the dendrite growth induced unstable SEI film (Lin et al., 2017). The reason for the Coulombic efficiency of VG/CC at initial several cycles is lower than 99% is due to the irreversible lithium that is consumed to form the SEI layer (Adams et al., 2018). After stable SEI film is formed, the Coulombic efficiency is increased higher than 99%. Benefiting from the large surface conductive area inducing reduced current density and the plentiful nucleation centers, the dendrites are inhibited by the designed VG/CC lithium host. The Li plating and stripping curves at various cycles of VG/CC and planar Cu are shown in Figures 5C,D, respectively. The voltage hysteresis of VG/CC is 67.3, 44.5, and 35.1 mV at 1, 50, and 100 cycles, respectively. For comparison, the voltage hysteresis of planar Cu is 85.3, 148.8, and 100.4 mV at 1, 50, and 100 cycles, respectively. Small voltage hysteresis indicates excellent plating/stripping behavior. Overpotential is another key parameter to evaluate the electrochemical performance of the lithium metal anode (Liu Y. et al., 2019). The nucleation overpotential is the difference between the lowest voltage point (nucleation potential) during discharge and the voltage platform after stabilization, which is used to overcome heterogeneous nucleation (Yan et al., 2016). The nucleation overpotential of VG/CC is 44.3 mV, which is much smaller than that of planar Cu (101.6 mV) in Figure S6. The lower overpotential means that lithium metal is more easily nucleated on the host. It is worth mentioning that the Li ions start to insert into the VG/CC above 0 V (Tian et al., 2018a). When the voltage continuous decreased below 0 V until the nucleation potential (indicated by the circle in Figure S6), the metallic lithium starts to nucleate and grow in the format of lithium metal (Tian et al., 2018b).

**Figure 6:**
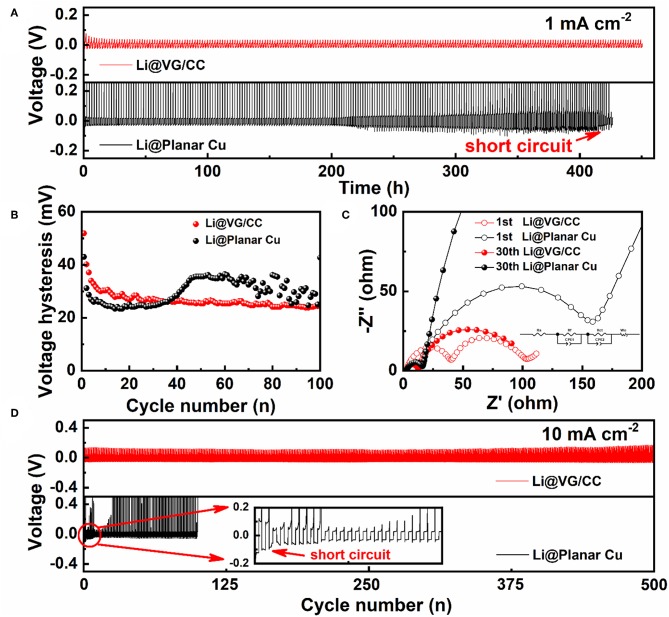
**(A)** Voltage profiles of the Li@VG/CC and Li@planar Cu and related **(B)** voltage hysteresis vs. cycle number profiles at a current density of 1 mA cm^−2^ with a fixed areal capacity of 1 mAh cm^−2^. **(C)** Nyquist plots of the Li@VG/CC and Li@planar Cu electrodes after the 1st and 30th cycles. **(D)** Long-time cycling stability test of the Li@VG/CC and Li@planar Cu electrodes at a current density of 10 mA cm^−2^ with a constant areal capacity of 1 mAh cm^−2^.

The electrochemical behavior of the Li@VG/CC and Li@planar Cu is evaluated by measuring their long-term cycling stability. The planar Cu and VG/CC are repeated plating/stripping at a fixed area capacity of 1 mAh cm^−2^ at a current density of 1 mA cm^−2^ without pre-store Li. The VG/CC exhibits a stable cycling performance until 450 h (Figure 6A). However, the voltage profile of the planar Cu, which exhibits a fluctuant trend from 200 h, may be due to the dendrite-induced short circuit. Figure 6B shows the voltage hysteresis profiles with cycles. The voltage hysteresis of VG/CC and planar Cu is reduced in the initial cycles. The voltage hysteresis of planar Cu rises to 35 mV after 50 cycles and starts to fluctuate. However, the voltage hysteresis of VG gradually stabilizes at 23–25 mV after 20 cycles. The charge transfer resistance (Rct) of VG/CC and planar Cu is measured by electrochemical impedance spectroscopy (EIS). An equivalent circuit model is used to simulate Rct as shown in the inset of Figure 6C. The charge transfer resistance of Li@planar Cu is decreased from 211.10 to 18.05 Ω from the 1st to 30th cycle. However, Li@VG/CC is decreased from 45.04 to 11.22 Ω, from the 1st to 30th cycle. Li@VG/CC electrode shows faster charge transfer resistance and a more stable interface (Kolosnitsyn et al., 2011). A long-term voltage cycle with a current density of 2 mA cm^−2^ is also measured in Figure S7. Figure 6D also shows the long-term cycle curve at a high current density of 10 mA cm^−2^. Li@planar Cu undergoes a random fluctuation during the cycling because the unstable interface of Li on Cu foil. Then the voltage hysteresis drops to ~92.4 mV abruptly after two cycles due to the dendrite-induced short circuit. However, Li@VG/CC can stably cycle over 500 cycles with a smaller voltage hysteresis (90.9 mV) and a small overpotential change. A comparison cycle performance of VG/CC with other 3D hosts is summarized in Table 1. The cycle stability of VG/CC is one of the best compared with the other state-of-art 3D hosts.

**Table 1:** Comparison of the performance of the VG/CC and other 3D Li metal hosts.

	**Current density (mA cm^**−2**^)**	**Area capacity (mAh cm^**−2**^)**	**Cycle number**	**References**
VG/CC	10	1	500	This work
Graphitized carbon fibers	2	1	300	Zuo et al., 2017
Cu foam @VG	3	3	50	Hu et al., 2019
3D porous Cu	2	1	120	Li et al., 2017
N doped GCF	3	1	900	Liu L. et al., 2018
3D ALD-CNTs	3	1	90	Zhang et al., 2017
CC/CNT	5	1	1,250	Liu F. et al., 2019
Oriented graphene foam (OGF)	10	0.5	250	Ma et al., 2018
Oxygen-CNT	4	1	200	Liu K. et al., 2018
Vertical grapheme nanowalls on Ni foam	0.5	1	500	Ren et al., 2018

**Figure 7:**
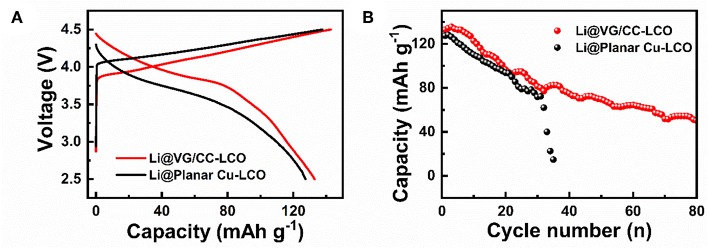
Electrochemical performance of a full battery with Li@VG/CC as the anode and LCO as the cathode: **(A)** charge-discharge curves at a current density of 0.2 C (1 C = 372 mAh g^−1^) in a potential range of 2.5–4.5 V, and **(B)** cycling performance at 0.2 C.

To further prove the practical application of Li@VG/CC, LCO is used as the cathode and Li@VG/CC as the anode to fabricate a full battery. Li@VG/CC is fabricated by depositing 2 mAh cm^−2^ of lithium metal into the VG/CC host as the anode. After electrochemical deposition of Li into VG/CC host, the cell is disassembled and reassembled with LCO as the cathode. Figure 7A shows the galvanostatic charge/discharge curves of the Li@VG/CC-LCO and Li@planar Cu-LCO at 0.2 C (1 C = 372 mAh g^−1^). The capacities of Li@VG/CC-LCO and Li@planar Cu-LCO are 133 and 127 mAh g^−1^, respectively. Figure 7B is cycle performance of the Li@VG/CC-LCO and Li@planar Cu-LCO at 0.2 C. After 35 cycles, the capacity of Li@planar Cu-LCO decays rapidly down to 0 mAh g^−1^. However, Li@VG/CC-LCO still maintains a capacity of 50 mAh g^−1^ at 80 cycles. Compared to planar Cu, the capacity and the stability are significantly improved.

## Conclusions

In summary, a conductive metal-free carbon-based VG/CC 3D porous structure is synthesized and used as the lithium metal host. The abundant void between VG nanosheets fully accommodates the lithium volume change and plays a significant role in inhibiting the formation of lithium dendrites. Our designed Li@VG/CC anode exhibits excellent cycle stability, high and stable Coulombic efficiency and a relatively small voltage hysteresis. Importantly, a full battery composed of Li@VG/CC as the anode and the LCO as the cathode exhibits high capacity of 133 mAh g^−1^ at 0.2 C. This special designed Li@VG/CC provides a new way as high-capacity lithium metal anode.

## Data Availability Statement

The raw data supporting the conclusions of this manuscript will be made available by the authors, without undue reservation, to any qualified researcher.

## Author Contributions

YW developed the concept and designed the experiments. CY and CM conducted the experiments. TX, JZ, and JX built the cells and carried out the performance characterizations. YW and XL co-supervised the research. CK, DK, and YS revised the work critically for important intellectual content. All authors discussed the results and co-wrote the manuscript.

### Conflict of Interest

The authors declare that the research was conducted in the absence of any commercial or financial relationships that could be construed as a potential conflict of interest.
