# Supplementary material for: Dendrite-Free Li Metal Plating/Stripping Onto Three-Dimensional Vertical-Graphene@Carbon-Cloth Host
Source: Front Chem. 2019 Oct 25;7:714. doi: 10.3389/fchem.2019.00714 (PMC6824185; doi:10.3389/fchem.2019.00714)
Supplement: Supplementary file 1 [file Table_1.docx]

Supporting Information

**Dendrite-free Li metal plating/stripping onto three-dimensional vertical-graphene@carbon-cloth host**

Congcong Yan^1^, Tingting Xu^1^, Caiyun Ma^1^, Jinhao Zang^1^, Junmin Xu^1^, Yumeng Shi^2^, Dezhi Kong^1^, Chang Ke^3^, Xinjian Li^1^, Ye Wang^1,^*

- - - 1. Key Laboratory of Material Physics of Ministry of Education, School of Physics and Engineering, Zhengzhou University, Zhengzhou 450052, China
      2. International Collaborative Laboratory of 2D Materials for Optoelectronics Science and Technology of Ministry of Education, Engineering Technology Research Center for 2D Material Information Function Devices and Systems of Guangdong Province, Institute of Microscale Optoelectronics, Shenzhen University, Shenzhen, 518060, China
      3. School of Electrical and Electronic Engineering, Nanyang Technological University, Nanyang Avenue, 639798, Singapore

*Corresponding author. Tel.: +86-18236756639. E-mail address: [wangye@zzu.edu.cn](mailto:wangye@zzu.edu.cn)


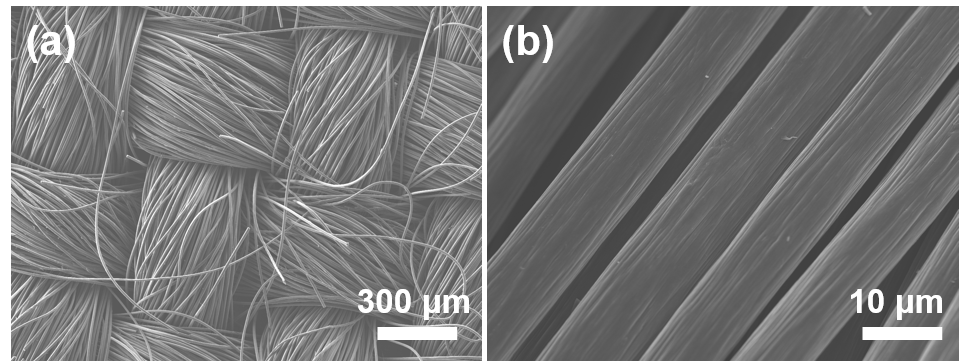


**FIGURE S1∣**SEM images of CC at **(a)** low and **(b)** high magnifications.


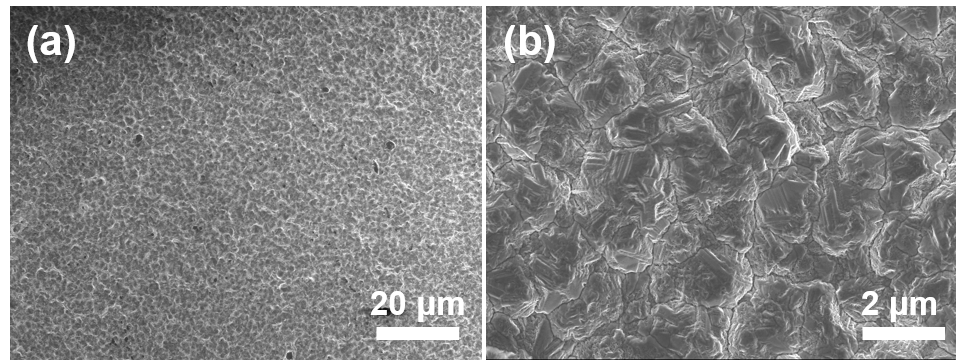


**FIGURE S2∣**SEM images of planar Cu at **(a)** low and **(b)** high magnifications.


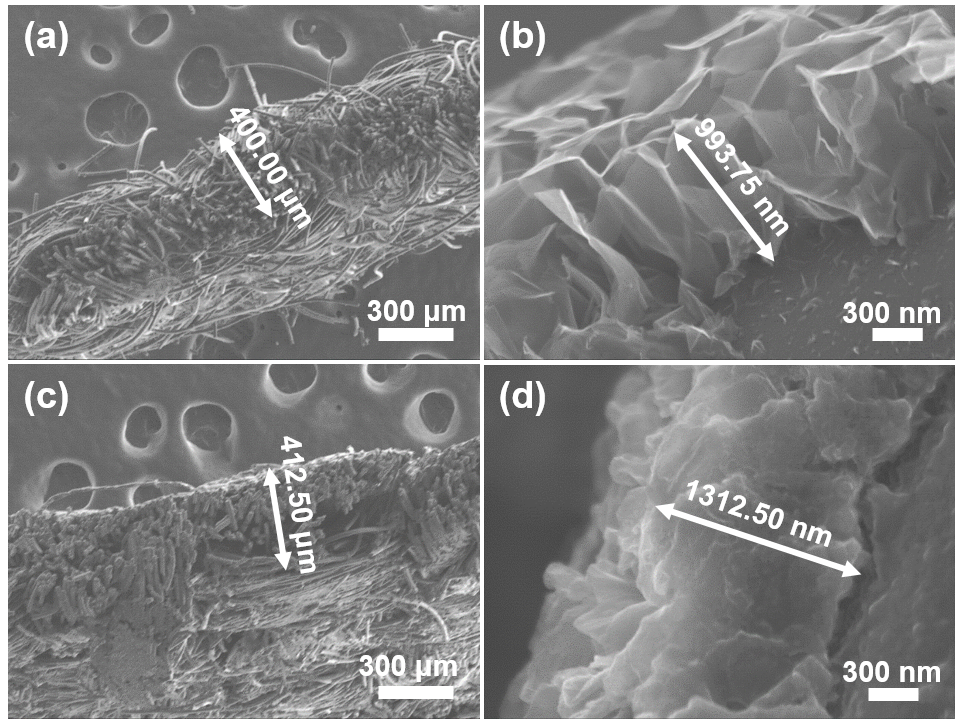


**FIGURE S3∣**Cross-sectional SEM images of **(a,b)** pristine VG/CC and **(c,d)** after Li deposition with a capacity of 4 mAh cm^-2^.

**
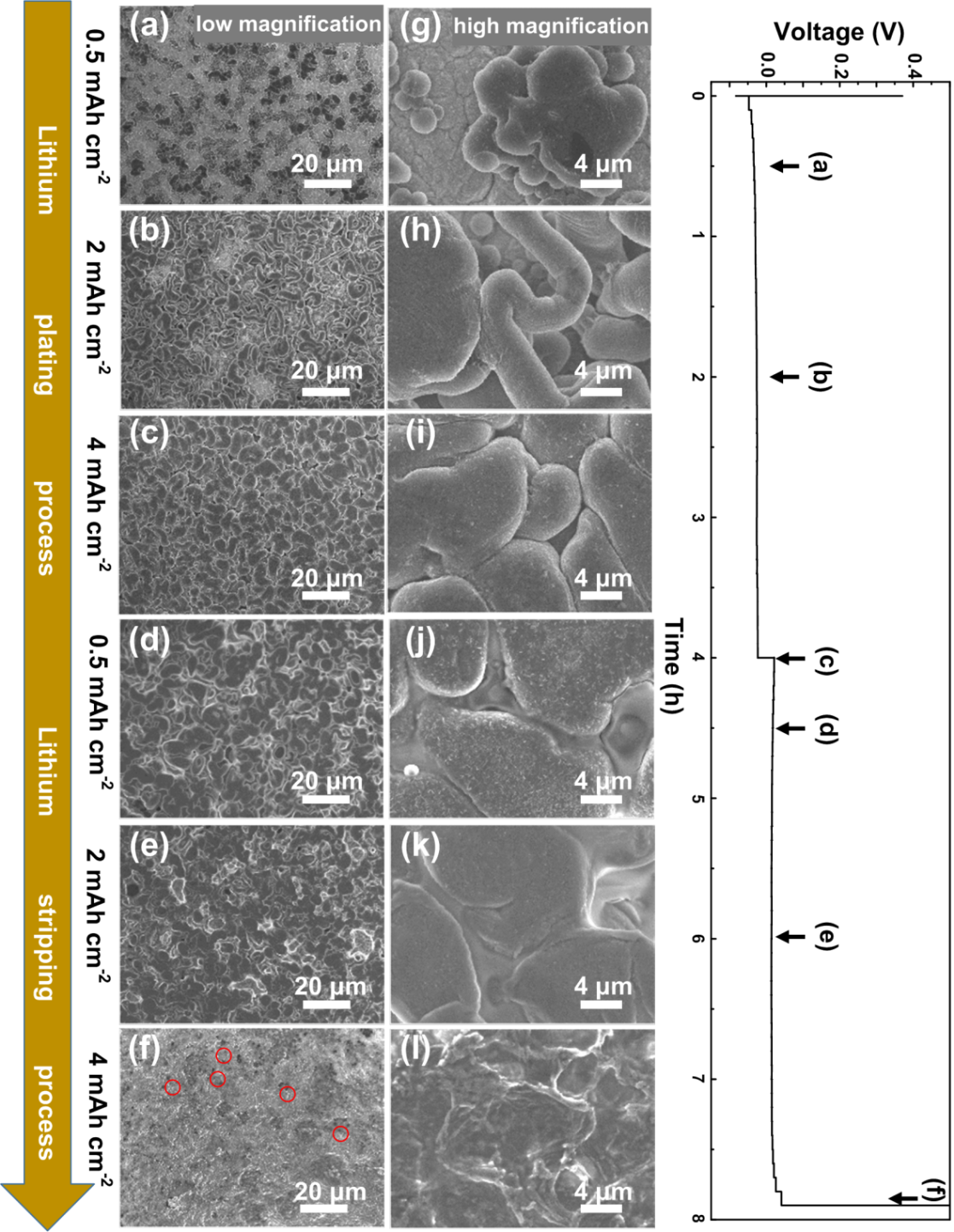
**

**FIGURE S4∣**Morphological evolution of Li metal plating and stripping on planar Cu. SEM images of planar Cu electrode after plated at a capacity of **(a, g)** 0.5 mAh cm^-2^ **(b, h)** 2 mAh cm^-2^ and **(c, i)** 4 mAh cm^-2^, and then stripped **(d, j)** 0.5 mAh cm^-2^, **(e, k)** 2 mAh cm^-2^ and **(f, l)** 4 mAh cm^-2^ at a current density of 1 mA cm^-2^.

**

**

**FIGURE S5∣**Coulombic efficiency of the Li@VG/CC, Li@planar Cu electrodes at a current density of 1 mA cm^-2^ with a constant areal capacity of 2 mAh cm^-2^.

**
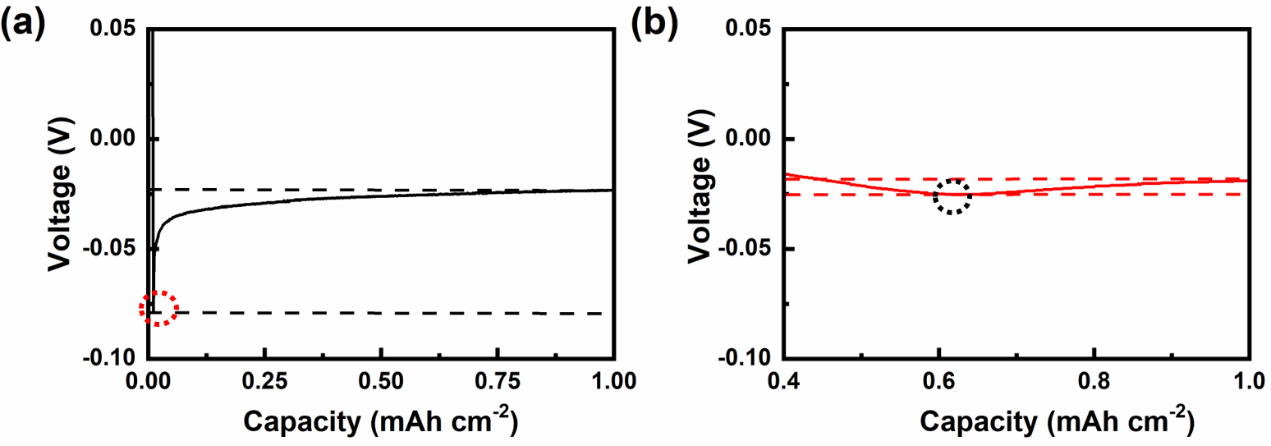
**

**FIGURE S6∣**Overpotential during Li deposition on **(a)** planar Cu and **(b)** VG/CC electrodes.


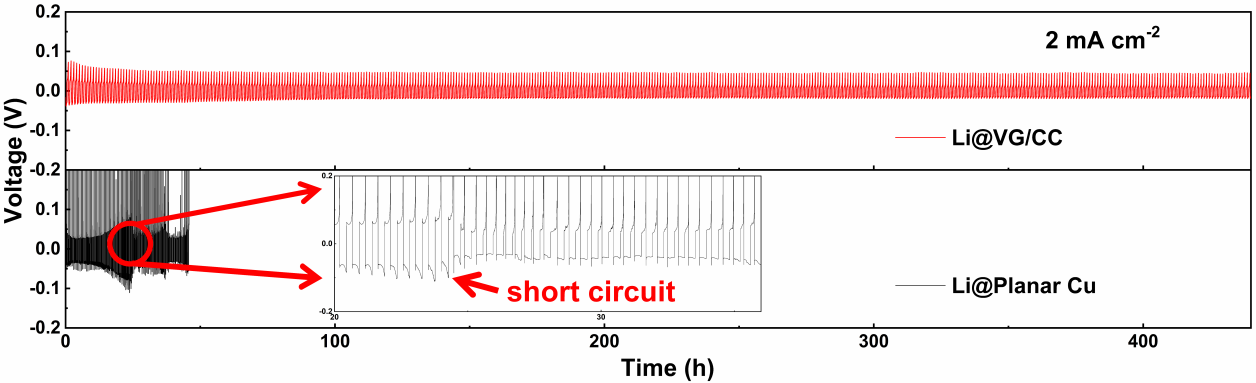


**FIGURE S7∣**Long-time cycling stability test of the Li@VG/CC and Li@planar Cu electrodes at a current density of 2 mA cm^-2^ with a constant areal capacity of 1 mAh cm^-2^.
